# Supplementary material for: Structural Analysis of the Novel Variants of SARS-CoV-2 and Forecasting in North America
Source: Viruses. 2021 May 17;13(5):930. doi: 10.3390/v13050930 (PMC8156069; doi:10.3390/v13050930)
Supplement: Supplementary file 1 [file viruses-13-00930-s001.zip › viruses-1189296-SMv2.pdf]

Supplementary Materials

Structural analysis of the novel variants of SARS-CoV-2 and forecasting in North America

Authors: Elena Quinonez[1], Majid Vahed[2], Abdolrazagh Hashemi Shahraki[2], and Mehdi Mirsaeidi[2*]

Table S1. Alteration of some amino acids of SARS-CoV-2 variants are presented relative to ancestral sequence of the Wuhan isolate

Variants	**Amino acid positions relative to ancestral sequence of the Wuhan isolate**																			
	**69**	**70**	**80**	**144**	**215**	**242**	**243**	**244**	**417**	**452**	**484**	**501**	**570**	**614**	**681**	**701**	**716**	**982**	**1118**	**1176**
**Wild type**	H	V	D	-	D	L	A	L	K	L	E	N	A	G	P	A	T	S	D	V
**B.1.1.7**	-	-	*	-	*	*	*	*	*	*	*	Y	D	*	H	*	I	A	H	*
**B.1.351**	*	*	A	Y	G	-	-	-	N	*	K	Y	*	*	*	V	*	*	*	*
**P.1**	*	*	*	Y	*	*	*	*	*	*	K	*	*	*	*	*	*	*	*	*
**B.1.617**	*	*	*	Y	*	*	*	*	*	R	Q	*	*	*	R	*	*	*	*	*

PNITNLCPFGEVFNATRFASVYAWNRKRISNCVADYSVLYNSASFSTFKCYGVSPTKLNDLCFT
NVYADSFVIRGDEVRQIAPGQTGKIADYNYKLPDDFTGCVIAWNSNNLDSKVGGNYNYLYRLF
RKSNLKPFERDISTEIYQAGSTPCNGVEGFNCYFPLQSYGFQPTNGVGYQPYRVVVLSFELLH
APATVCGPKKSTN

417 484 501

Figure S1. Sequence alignment of S-RBD of SARS-CoV-2 genes with mutations K417N, E484K, N501Y

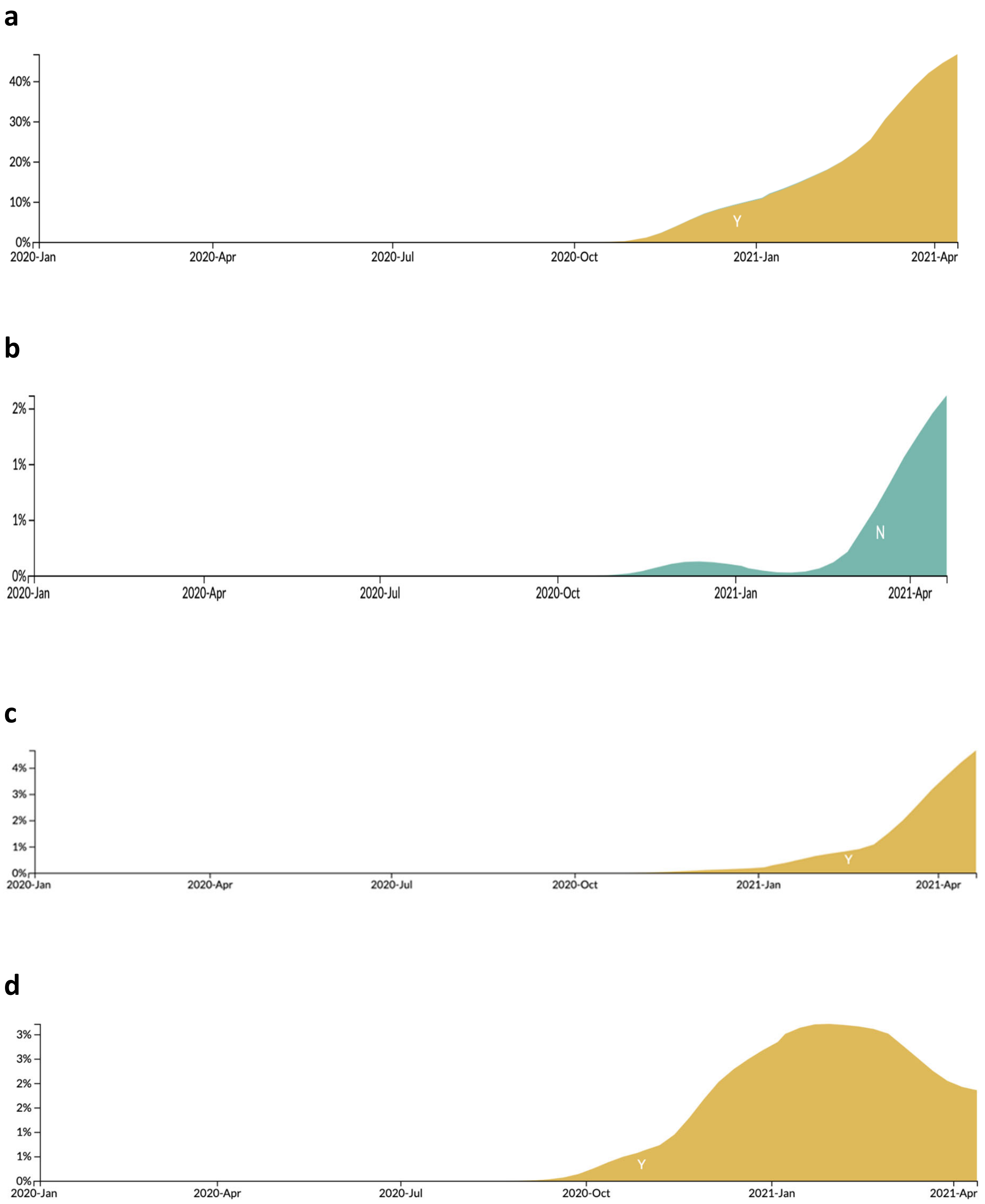

Figure S2: Frequency of novel variants of SARS-CoV 2 over time collected from North American subsample. a) B.1.1.7, b) B.1.617, c) P.1 and d) B.1.351

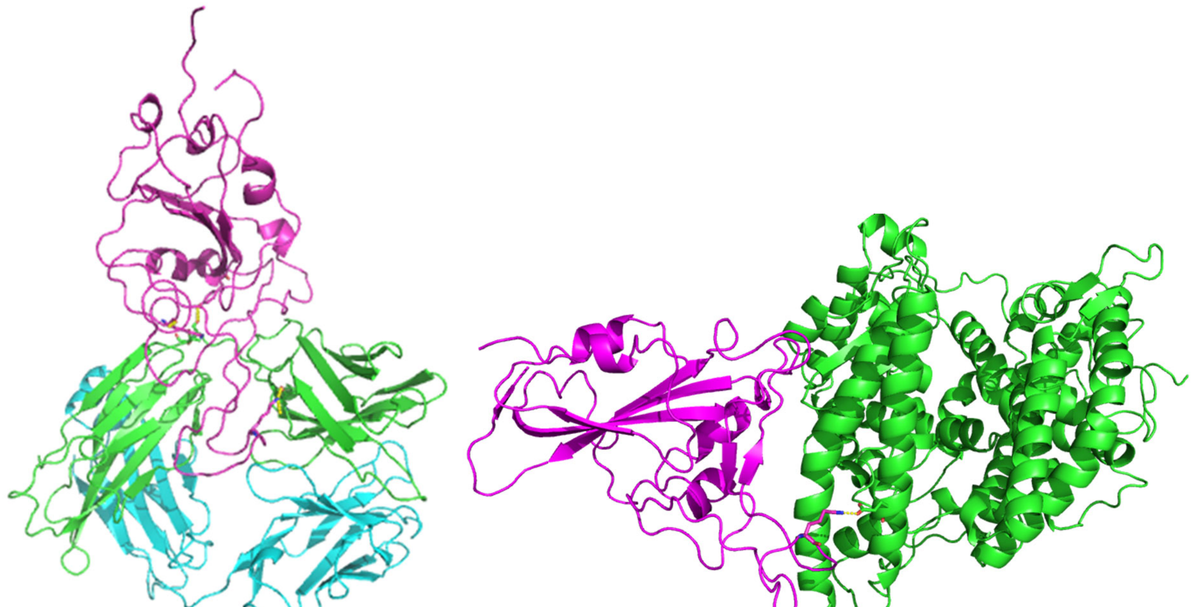

Figure S3: Docking structures of the interaction of E484K mutant of SARS-CoV-2 S-RBD protein in complex with a potent neutralizing CV30 antibody/hACE-2.

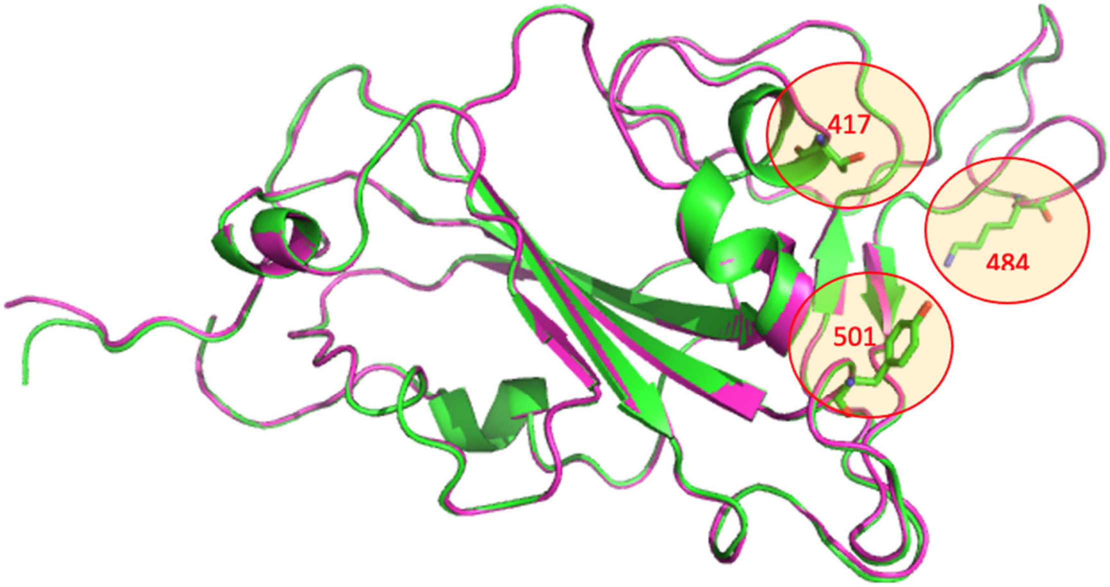

Figure S4. Superposition of wild/mutant SARS-CoV-2 S-RBD protein structures. The red rings in the photo indicate the three mutation points in the S-RBD that are in the flexible coil region. The structure displayed in purple indicates wild and the green color indicates mutated.

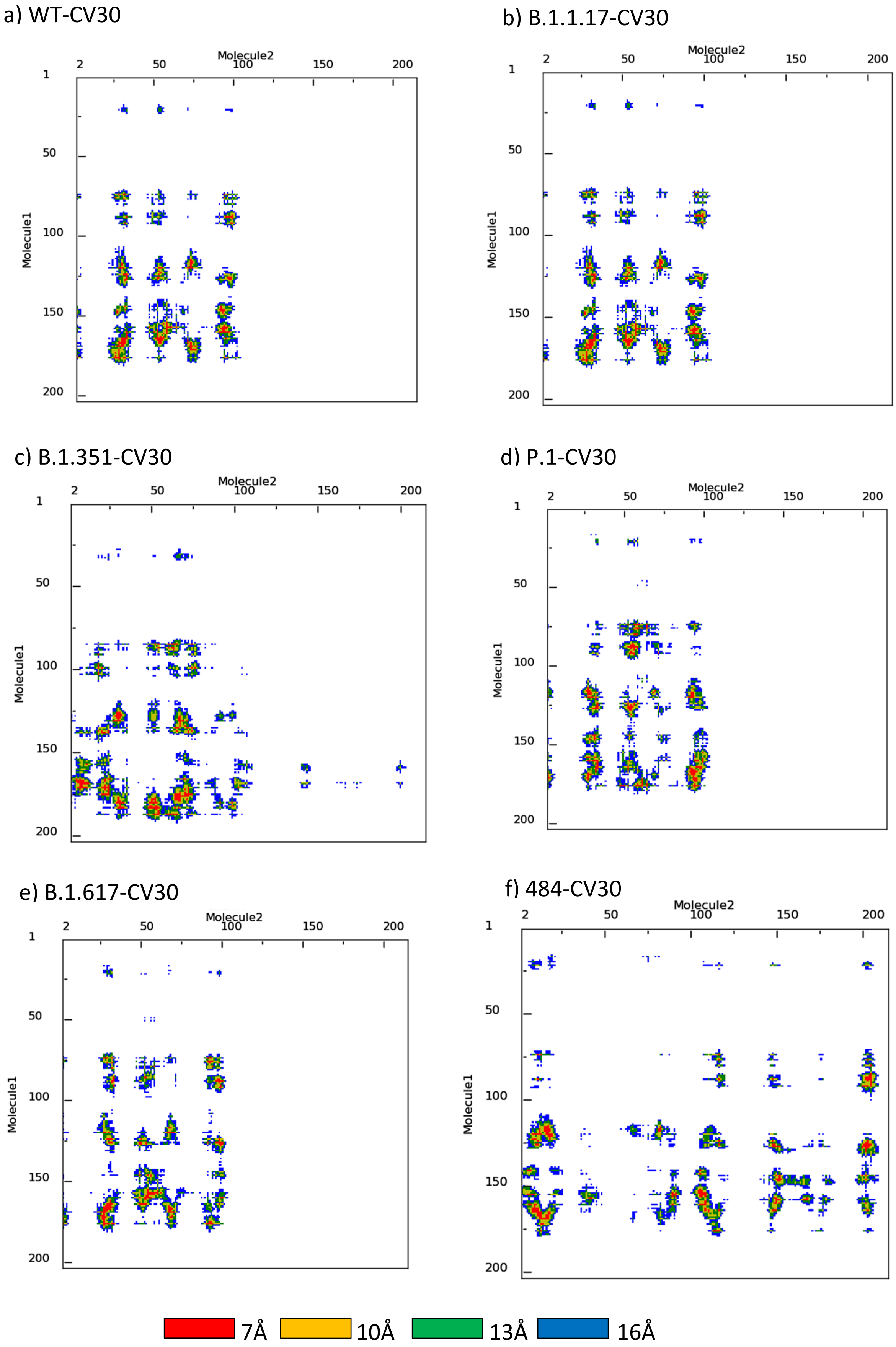
a) WT-CV30
b) B.1.1.17-CV30
c) B.1.351-CV30
d) P.1-CV30
e) B.1.617-CV30
f) 484-CV30
Molecule2
Molecule1
2
50
100
150
200
1
7Å
10Å
13Å
16Å

Figure S5: COCOMAPS distance contact maps for Interaction of different variants of SARS-CoV 2 spike with neutralizing antibody (CV30)/ACE-2. The color codes are reported in the legends under the maps.

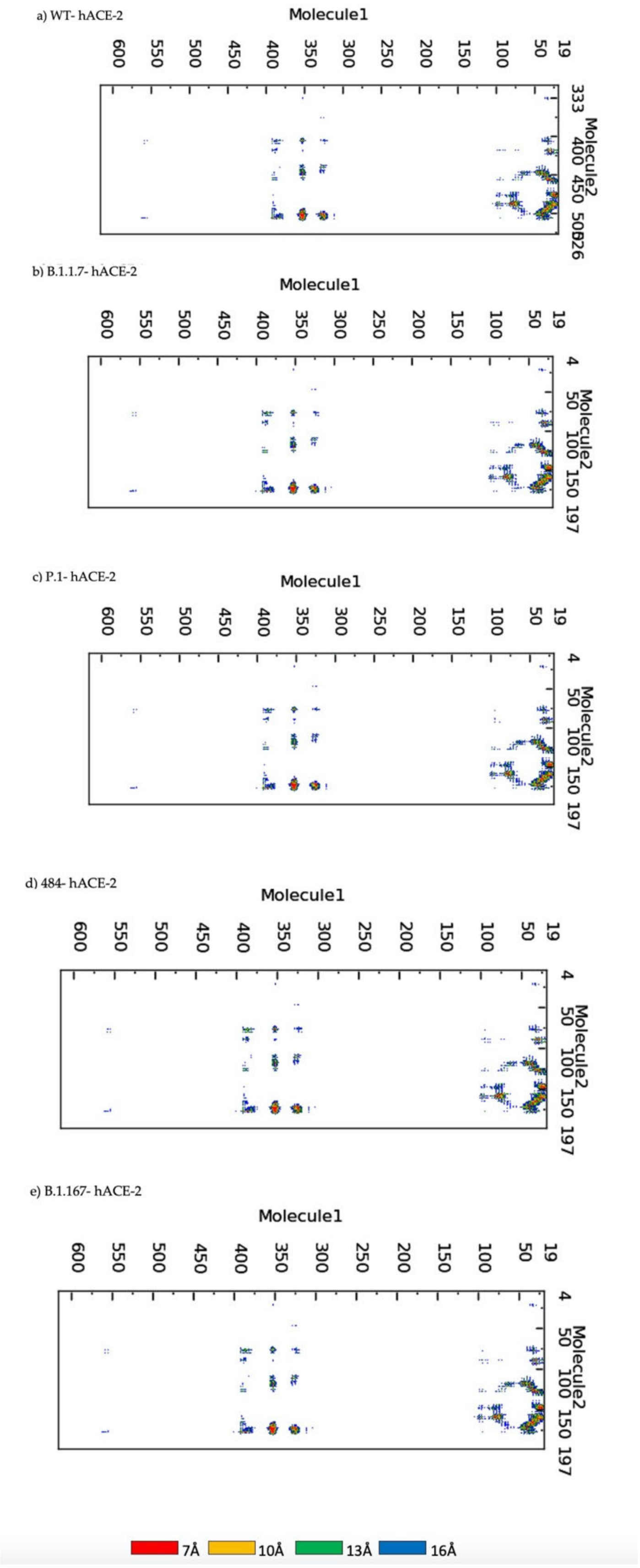
a) WT- hACE-2
Molecule1
Molecule2
b) B.1.1.7- hACE-2
Molecule1
Molecule2
c) P.1- hACE-2
Molecule1
Molecule2
d) 484- hACE-2
Molecule1
Molecule2
e) B.1.167- hACE-2
Molecule1
Molecule2
7Å
10Å
13Å
16Å

Figure S5: COCOMAPS distance contact maps for Interaction of different variants of SARS-CoV-2 spike with neutralizing hACE-2. The color codes are reported in the legends under the maps.
